# Poly[[(1,10-phenanthroline)(μ-l-tartrato)zinc] hexa­hydrate]

**DOI:** 10.1107/S1600536811024780

**Published:** 2011-06-30

**Authors:** Gui-Ying Dong, Cui-Hong He, Tong-Fei Liu, Guang-Hua Cui, Xiao-Chen Deng

**Affiliations:** a College of Chemical Engineering, Hebei United University, Tangshan 063009, People’s Republic of China; b Qian’an College, Hebei United University, Tangshan 063009, People’s Republic of China

## Abstract

The title compouand {[Zn(C_4_H_4_O_6_)(C_12_H_8_N_2_)]·6H_2_O}_*n*_, has a linear chain structure parallel to [100] with Zn(C_4_H_4_O_6_)(C_12_H_8_N_2_) repeat units; the asymmetric unit consists of one Zn^2+^ cation, one l-tartrate dianion, one 1,10-phenanthroline and six free water mol­ecules. The Zn atom is in a distorted octa­hedral ZnN_2_O_4_ coordination environment. The crystal structure is stabilized by O—H⋯O hydrogen bonds and π–π stacking of the phenanthroline units [centroid–centroid distances in the range 3.552 (2)–3.625 (2)Å] occurs between the chains. The title compound is isotypic with the Cu and Mn analogues.

## Related literature

For chiral multifunctional materials constructed from tartrate, see: Liu *et al.* (2008 ▶, 2010 ▶); Gelbrich *et al.* (2006 ▶); Kitagawa *et al.* (2004 ▶); Ma *et al.* (2007 ▶); Adama *et al.* (2007 ▶); Lin *et al.* (2009 ▶); Templeton *et al.* (1985 ▶). For the isotypic copper and manganese analogues, see: McCann *et al.* (1997 ▶); Zhang *et al.* (2003 ▶). 
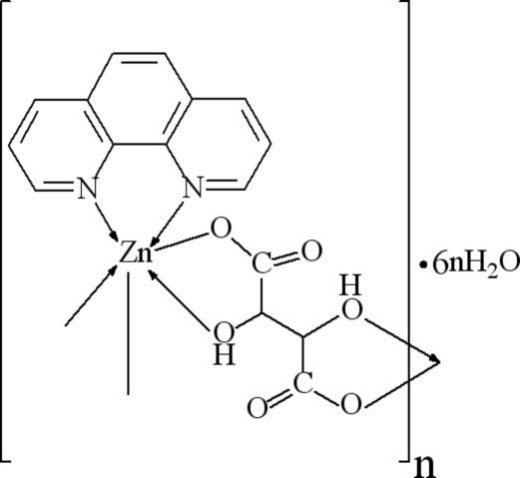


## Experimental

### 

#### Crystal data

[Zn(C_4_H_4_O_6_)(C_12_H_8_N_2_)]·6H_2_O
*M* *_r_* = 501.76
Orthorhombic, 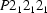

*a* = 6.632 (2) Å
*b* = 15.301 (4) Å
*c* = 20.087 (5) Å
*V* = 2038.4 (10) Å^3^
*Z* = 4
Mo *K*α radiation
μ = 1.27 mm^−1^
*T* = 295 K
0.25 × 0.18 × 0.16 mm

#### Data collection

Bruker SMART CCD area-detector diffractometer
Absorption correction: multi-scan (*SADABS*; Sheldrick, 1996 ▶) *T*_min_ = 0.754, *T*_max_ = 0.844
16280 measured reflections
3541 independent reflections
3251 reflections with *I* > 2σ(*I*)
*R*_int_ = 0.036

#### Refinement

*R*[*F* ^2^ > 2σ(*F* ^2^)] = 0.027
*wR*(*F* ^2^) = 0.071
*S* = 0.96
3541 reflections
280 parameters
3 restraints
H-atom parameters constrained
Δρ_max_ = 0.32 e Å^−3^
Δρ_min_ = −0.29 e Å^−3^
Absolute structure: Flack (1983 ▶), 1456 Friedel pairs
Flack parameter: 0.025 (12)

### 

Data collection: *SMART* (Bruker, 1998 ▶); cell refinement: *SAINT* (Bruker, 1998 ▶); data reduction: *SAINT*; program(s) used to solve structure: *SHELXS97* (Sheldrick, 2008 ▶); program(s) used to refine structure: *SHELXL97* (Sheldrick, 2008 ▶); molecular graphics: *SHELXTL* (Sheldrick, 2008 ▶); software used to prepare material for publication: *SHELXTL*.

## Supplementary Material

Crystal structure: contains datablock(s) I, global. DOI: 10.1107/S1600536811024780/rk2279sup1.cif

Structure factors: contains datablock(s) I. DOI: 10.1107/S1600536811024780/rk2279Isup2.hkl

Additional supplementary materials:  crystallographic information; 3D view; checkCIF report

## Figures and Tables

**Table 1:** Hydrogen-bond geometry (Å, °)

*D*—H⋯*A*	*D*—H	H⋯*A*	*D*⋯*A*	*D*—H⋯*A*
O1*W*—H1*A*⋯O5*W*^i^	0.85	1.99	2.787 (3)	157
O2*W*—H2*A*⋯O3*W*	0.85	2.05	2.819 (4)	150
O2*W*—H2*B*⋯O6*W*^ii^	0.85	1.98	2.822 (4)	172
O3*W*—H3*A*⋯O5^iii^	0.85	2.17	2.802 (3)	131
O3*W*—H3*B*⋯O1*W*^iv^	0.85	1.93	2.776 (4)	177
O4*W*—H4*A*⋯O5*W*^i^	0.85	2.00	2.789 (4)	154
O4*W*—H4*B*⋯O6^v^	0.85	1.99	2.718 (3)	143
O5*W*—H5*A*⋯O3^iii^	0.85	2.04	2.880 (3)	168
O5*W*—H5*B*⋯O1	0.85	2.10	2.909 (3)	160
O6*W*—H6*A*⋯O1*W*^vi^	0.85	2.02	2.816 (4)	155
O6*W*—H6*B*⋯O5	0.85	2.04	2.886 (3)	174
O2—H21⋯O2*W*^vii^	0.85	1.82	2.655 (3)	164
O4—H22⋯O4*W*^viii^	0.85	1.75	2.599 (3)	178

Symmetry codes: (i) 
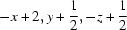
; (ii) 
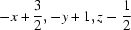
; (iii) 
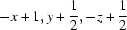
; (iv) 
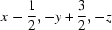
; (v) 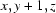; (vi) 
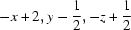
; (vii) 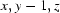; (viii) 
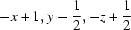
.

**Fig. 1.**
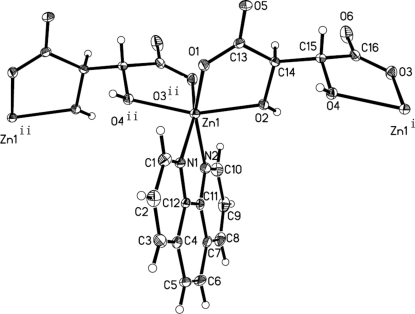
Part of the structure of I, showing the bridging mode of the L-tartrate anion and one of the independent structural units with the atom numbering scheme. Displacement ellipsoids are drawn at the 30% probability level. H atoms are presented as a small spheres of arbitrary radius. Symmetry codes: (i) x-1, y, z; (ii) x+1, y, z.

**Fig. 2.**
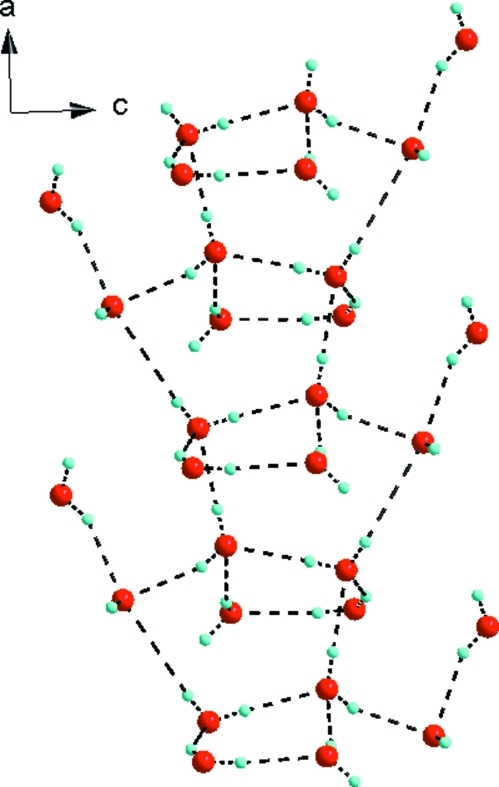
The one-dimensional helical chain water clusters in I.

## supplementary crystallographic information

### Comment

An enormous effort has been devoted to the development of new homochiral coordination polymer duo to their the possibility of applications to enantioselective separation, catalytic processes and magneto-optical processes (Kitagawa *et al.*, 2004; Ma *et al.*, 2007; Liu *et al.*, 2008; Gelbrich *et al.*, 2006)). L-tartaric acid, a simple and inexpensive chiral ligand source, was often used to construct novel chiral multifunctional metal-organic frameworks (McCann *et al.*,1997; Zhang *et al.*, 2003). However, only four zinc-tartrate compounds have been reported (Adama *et al.*, 2007; Lin *et al.*, 2009; Liu *et al.*, 2010; Templeton *et al.*, 1985) up to now. We report here the synthesis and crystal structure of the first mixed-ligand zinc(II) complex with tartrate and 1,10-phenanthroline, I, which has a linear chain structure.

The asymmetric unit of I consists of one Zn atom, one L-tartrate dianion, one phenanthroline and six free water molecules, (Fig. 1). The Zn atom is hexacoordinated by four O atoms (Zn-O = 2.046 (2)-2.200 (2)Å) and two N atoms (Zn-N = 2.127 (2)Å and 2.132 (2)Å) forming [ZnO_4_N_2_] distorted octahedral geometry with three *trans*-angles form 159.21 (10)° to 160.73 (10)°. The Zn-*O*(hydroxy) and Zn–*O*(carboxylate) distances are typical for Zn-O bonds. The L-tartrate dianion adopts η_4_µ_2_-chelating/bridging mode to extend Zn(C_12_H_8_N_2_)^2+^ to one-dimensional polymeric chain, which is assembled together to genterate a zipper-like double chain through strong π–π packing interactions between parallel phenanthroline aromatic rings with centroid–centroid distances of 3.552 (2)-3.625 (2)Å. The most noteworthy structural feature of I, is the existence of one-dimensional helical chain water cluster (Fig. 2). In crystal achitecture of I, there are six crystllography independent lattice water molecules, which are interconnected by hydrogen bonds forming a right-handed helical chain. The average Ow···Ow distance of 2.79 (2)Å in I is slightly small than the Ow···Ow distance observed in liquid water (2.85 (3)Å). The hydrogen bonding interactions between the Ow atoms from water cluster chains and the tartrate O atoms from one-dimensional double zipper chains with the average Ow···O distance of 2.77 (2)Å lead to the formation of a three-dimensional supramolecular framework of I.

### Experimental

A mixture of Zn(NO_3_)_2_^.^6H_2_O (298 mg, 1 mmol), L-(+)-tartaric acid (150 mg, 1 mmol) and 1,10-phenanthroline (180 mg, 1 mmol) in H_2_O (12 ml) was placed in a teflon-lined stainless vessel and heated to 413 K for 72 h. Then, the reaction system was cooled to room temperature during 24 h to give rise to colourless crystals, which were collected and washed with water. Yield 0.191 g (38% based on Zn). Analysis calculated for C_16_H_24_N_2_ZnO_12_ (501.76): C 38.30, H 4.82, N 5.58%; found: C 38.06, H 4.71 N 5.49%.

### Refinement

H atoms bonded to C atoms were placed at calculated idealized positions using a riding model - C–H = 0.93%A for 1,10-phenanthroline and 0.98%A for L-tartrate, and *U*_iso_(H) = 1.2*U*_eq_(C). Water H atoms were located in a difference Fourier map and refined isotropically, with O–H and H···H distance restraints of 0.85 (1) and 1.37 (1)Å, respectively.

### Crystal data

**Table:**

[Zn(C_4_H_4_O_6_)(C_12_H_8_N_2_)]·6H_2_O	*F*(000) = 1040
*M**_r_* = 501.76	*D*_x_ = 1.635 Mg m^−^^3^
Orthorhombic, *P*2_1_2_1_2_1_	Mo *K*α radiation, λ = 0.71073 Å
Hall symbol: P 2ac 2ab	Cell parameters from 3086 reflections
*a* = 6.632 (2) Å	θ = 4.3–23.8°
*b* = 15.301 (4) Å	µ = 1.27 mm^−^^1^
*c* = 20.087 (5) Å	*T* = 295 K
*V* = 2038.4 (10) Å^3^	Block, colourless
*Z* = 4	0.25 × 0.18 × 0.16 mm

### Data collection

**Table:**

Bruker SMART CCD area-detector diffractometer	3541 independent reflections
Radiation source: fine-focus sealed tube	3251 reflections with *I* > 2σ(*I*)
graphite	*R*_int_ = 0.036
φ and ω scans	θ_max_ = 25.0°, θ_min_ = 1.7°
Absorption correction: multi-scan (*SADABS*; Sheldrick, 1996)	*h* = −7→7
*T*_min_ = 0.754, *T*_max_ = 0.844	*k* = −18→18
16280 measured reflections	*l* = −23→23

### Refinement

**Table:**

Refinement on *F*^2^	Secondary atom site location: difference Fourier map
Least-squares matrix: full	Hydrogen site location: inferred from neighbouring sites
*R*[*F*^2^ > 2σ(*F*^2^)] = 0.027	H-atom parameters constrained
*wR*(*F*^2^) = 0.071	*w* = 1/[σ^2^(*F*_o_^2^) + (0.0466*P*)^2^ + 0.136*P*] where *P* = (*F*_o_^2^ + 2*F*_c_^2^)/3
*S* = 0.96	(Δ/σ)_max_ = 0.001
3541 reflections	Δρ_max_ = 0.32 e Å^−^^3^
280 parameters	Δρ_min_ = −0.29 e Å^−^^3^
3 restraints	Absolute structure: Flack (1983), 1456 Friedel pairs
Primary atom site location: structure-invariant direct methods	Flack parameter: 0.025 (12)

### Special details

**Table:**

Geometry. All s.u.'s (except the s.u. in the dihedral angle between two l.s. planes) are estimated using the full covariance matrix. The cell s.u.'s are taken into account individually in the estimation of s.u.'s in distances, angles and torsion angles; correlations between s.u.'s in cell parameters are only used when they are defined by crystal symmetry. An approximate (isotropic) treatment of cell s.u.'s is used for estimating s.u.'s involving l.s. planes.
Refinement. Refinement of *F*^2^ against ALL reflections. The weighted *R*-factor *wR* and goodness of fit *S* are based on *F*^2^, conventional *R*-factors *R* are based on *F*, with *F* set to zero for negative *F*^2^. The threshold expression of *F*^2^ > σ(*F*^2^) is used only for calculating *R*-factors(gt) *etc*. and is not relevant to the choice of reflections for refinement. *R*-factors based on *F*^2^ are statistically about twice as large as those based on *F*, and *R*-factors based on ALL data will be even larger.

### Fractional atomic coordinates and isotropic or equivalent isotropic displacement parameters (Å^2^)

**Table:**

	*x*	*y*	*z*	*U*_iso_*/*U*_eq_	
O3	0.1075 (3)	−0.01758 (12)	0.20025 (11)	0.0366 (5)	
C16	0.2759 (4)	−0.02548 (17)	0.22862 (14)	0.0298 (6)	
O4	0.3195 (3)	0.12657 (10)	0.20244 (9)	0.0284 (4)	
C15	0.3940 (4)	0.05857 (18)	0.24324 (14)	0.0250 (6)	
H15	0.3646	0.0748	0.2894	0.030*	
Zn1	1.00853 (5)	0.102577 (17)	0.169795 (13)	0.02690 (10)	
C12	1.0062 (5)	0.22189 (16)	0.05463 (11)	0.0271 (5)	
O5W	1.0162 (4)	0.30592 (14)	0.32821 (11)	0.0563 (6)	
N2	1.0235 (4)	0.06704 (14)	0.06729 (11)	0.0314 (5)	
N1	1.0013 (4)	0.22682 (12)	0.12231 (9)	0.0275 (4)	
C4	1.0048 (5)	0.29597 (17)	0.01369 (13)	0.0338 (6)	
C11	1.0136 (5)	0.13644 (17)	0.02535 (12)	0.0287 (5)	
O1	0.8915 (3)	0.14883 (13)	0.25782 (9)	0.0361 (5)	
O2	0.6953 (3)	0.05600 (11)	0.17118 (9)	0.0304 (4)	
C5	1.0043 (5)	0.2840 (2)	−0.05749 (13)	0.0422 (7)	
H5	1.0039	0.3328	−0.0851	0.051*	
C1	0.9968 (5)	0.30549 (16)	0.14973 (13)	0.0353 (6)	
H1	0.9926	0.3097	0.1959	0.042*	
C3	1.0025 (5)	0.37765 (18)	0.04495 (16)	0.0431 (7)	
H3	1.0040	0.4286	0.0197	0.052*	
C6	1.0043 (6)	0.2046 (2)	−0.08455 (13)	0.0466 (7)	
H6	0.9991	0.1992	−0.1306	0.056*	
C14	0.6229 (4)	0.04665 (19)	0.23801 (14)	0.0265 (6)	
H14	0.6551	−0.0127	0.2529	0.032*	
C2	0.9980 (5)	0.38216 (16)	0.11246 (16)	0.0425 (7)	
H2	0.9957	0.4362	0.1337	0.051*	
C7	1.0121 (5)	0.12713 (19)	−0.04425 (13)	0.0367 (6)	
C8	1.0180 (6)	0.0416 (2)	−0.06949 (15)	0.0472 (8)	
H8	1.0127	0.0323	−0.1152	0.057*	
O4W	0.6195 (4)	0.77423 (16)	0.23686 (14)	0.0661 (8)	
C9	1.0313 (5)	−0.0270 (2)	−0.02751 (17)	0.0488 (9)	
H9	1.0393	−0.0836	−0.0443	0.059*	
O6	0.3450 (3)	−0.09500 (13)	0.24989 (13)	0.0541 (6)	
O6W	0.9593 (4)	0.09008 (17)	0.44919 (13)	0.0668 (8)	
O5	0.6820 (3)	0.11830 (13)	0.34059 (9)	0.0421 (5)	
C13	0.7393 (4)	0.11022 (17)	0.28205 (13)	0.0284 (6)	
O2W	0.5435 (5)	0.93830 (16)	0.08799 (12)	0.0691 (9)	
C10	1.0329 (5)	−0.0129 (2)	0.04130 (16)	0.0433 (8)	
H10	1.0407	−0.0607	0.0697	0.052*	
O3W	0.4092 (5)	0.76383 (18)	0.08031 (13)	0.0717 (9)	
O1W	0.8120 (4)	0.74506 (16)	0.05396 (12)	0.0688 (8)	
H2A	0.5009	0.8888	0.1012	0.103*	
H4A	0.7053	0.7907	0.2081	0.103*	
H4B	0.5115	0.8013	0.2270	0.103*	
H6B	0.8707	0.0962	0.4190	0.103*	
H6A	0.9938	0.1431	0.4439	0.103*	
H5A	0.9930	0.3578	0.3153	0.103*	
H2B	0.5516	0.9338	0.0459	0.103*	
H5B	0.9921	0.2536	0.3163	0.103*	
H1A	0.8804	0.7738	0.0823	0.103*	
H1B	0.6869	0.7508	0.0620	0.103*	
H3B	0.3756	0.7608	0.0396	0.103*	
H3A	0.3169	0.7389	0.1028	0.103*	
H21	0.6634	0.0119	0.1475	0.103*	
H22	0.3426	0.1744	0.2228	0.103*	

### Atomic displacement parameters (Å^2^)

**Table:**

	*U*^11^	*U*^22^	*U*^33^	*U*^12^	*U*^13^	*U*^23^
O3	0.0324 (12)	0.0319 (10)	0.0456 (12)	−0.0066 (8)	−0.0060 (10)	0.0044 (9)
C16	0.0262 (16)	0.0296 (14)	0.0336 (15)	−0.0017 (11)	0.0054 (12)	0.0052 (12)
O4	0.0266 (10)	0.0255 (9)	0.0330 (10)	0.0005 (8)	−0.0037 (8)	0.0036 (8)
C15	0.0249 (15)	0.0290 (13)	0.0211 (15)	−0.0003 (11)	0.0004 (12)	0.0035 (12)
Zn1	0.02568 (16)	0.03218 (15)	0.02283 (15)	0.00036 (16)	0.00039 (16)	0.00170 (11)
C12	0.0165 (12)	0.0402 (12)	0.0246 (12)	0.0009 (15)	−0.0026 (14)	0.0047 (10)
O5W	0.0625 (16)	0.0543 (12)	0.0520 (13)	0.0103 (13)	−0.0063 (17)	0.0013 (10)
N2	0.0297 (14)	0.0366 (11)	0.0279 (11)	−0.0001 (11)	0.0019 (12)	−0.0022 (9)
N1	0.0238 (11)	0.0356 (10)	0.0230 (10)	0.0007 (12)	0.0002 (12)	0.0012 (8)
C4	0.0178 (13)	0.0480 (14)	0.0355 (14)	0.0030 (16)	−0.0017 (16)	0.0086 (11)
C11	0.0167 (13)	0.0449 (13)	0.0247 (12)	−0.0039 (14)	−0.0001 (13)	−0.0012 (10)
O1	0.0322 (12)	0.0496 (12)	0.0263 (10)	−0.0117 (9)	0.0021 (9)	−0.0066 (9)
O2	0.0253 (10)	0.0444 (10)	0.0215 (10)	−0.0036 (8)	0.0049 (8)	−0.0063 (8)
C5	0.0266 (15)	0.0682 (19)	0.0317 (14)	−0.0002 (19)	−0.0007 (16)	0.0209 (13)
C1	0.0337 (15)	0.0380 (13)	0.0341 (13)	0.0063 (15)	−0.0014 (15)	−0.0038 (11)
C3	0.0305 (15)	0.0443 (15)	0.0545 (18)	0.0027 (17)	0.0012 (18)	0.0182 (13)
C6	0.0348 (17)	0.085 (2)	0.0199 (13)	−0.004 (2)	−0.0027 (17)	0.0078 (14)
C14	0.0231 (16)	0.0336 (14)	0.0229 (14)	0.0021 (11)	0.0023 (12)	0.0053 (12)
C2	0.0375 (16)	0.0327 (13)	0.0572 (19)	0.0038 (17)	−0.0030 (18)	−0.0016 (12)
C7	0.0215 (14)	0.0637 (17)	0.0250 (13)	−0.0066 (16)	−0.0028 (14)	−0.0034 (12)
C8	0.0355 (18)	0.075 (2)	0.0308 (15)	−0.006 (2)	−0.0018 (17)	−0.0196 (14)
O4W	0.0657 (18)	0.0476 (14)	0.0849 (19)	0.0199 (12)	0.0118 (15)	0.0257 (13)
C9	0.040 (2)	0.0525 (17)	0.054 (2)	−0.0013 (16)	−0.0003 (17)	−0.0231 (15)
O6	0.0400 (13)	0.0317 (11)	0.0906 (18)	0.0011 (10)	0.0008 (12)	0.0195 (12)
O6W	0.079 (2)	0.0599 (14)	0.0614 (16)	−0.0124 (15)	−0.0221 (14)	0.0128 (12)
O5	0.0411 (12)	0.0659 (14)	0.0193 (10)	−0.0092 (10)	0.0030 (9)	−0.0049 (9)
C13	0.0284 (15)	0.0348 (13)	0.0220 (14)	0.0016 (12)	−0.0018 (11)	−0.0002 (12)
O2W	0.099 (3)	0.0630 (14)	0.0455 (14)	−0.0279 (15)	0.0035 (15)	−0.0172 (11)
C10	0.042 (2)	0.0426 (15)	0.0450 (17)	−0.0001 (15)	0.0033 (16)	−0.0089 (13)
O3W	0.096 (2)	0.0713 (18)	0.0477 (15)	−0.0241 (15)	0.0004 (14)	0.0072 (13)
O1W	0.0779 (19)	0.0765 (17)	0.0521 (15)	0.0169 (15)	−0.0096 (14)	−0.0161 (13)

### Geometric parameters (Å, °)

**Table:**

O3—C16	1.260 (3)	C5—C6	1.331 (4)
O3—Zn1^i^	2.046 (2)	C5—H5	0.9300
C16—O6	1.235 (3)	C1—C2	1.392 (4)
C16—C15	1.534 (4)	C1—H1	0.9300
O4—C15	1.414 (3)	C3—C2	1.358 (4)
O4—Zn1^i^	2.1952 (19)	C3—H3	0.9300
O4—H22	0.8525	C6—C7	1.437 (4)
C15—C14	1.533 (4)	C6—H6	0.9300
C15—H15	0.9800	C14—C13	1.524 (4)
Zn1—O3^ii^	2.046 (2)	C14—H14	0.9800
Zn1—O1	2.0567 (19)	C2—H2	0.9300
Zn1—N1	2.1274 (19)	C7—C8	1.403 (4)
Zn1—N2	2.132 (2)	C8—C9	1.350 (5)
Zn1—O4^ii^	2.1952 (19)	C8—H8	0.9300
Zn1—O2	2.1966 (19)	O4W—H4A	0.8490
C12—N1	1.362 (3)	O4W—H4B	0.8507
C12—C4	1.400 (3)	C9—C10	1.399 (4)
C12—C11	1.434 (4)	C9—H9	0.9300
O5W—H5A	0.8493	O6W—H6B	0.8495
O5W—H5B	0.8502	O6W—H6A	0.8494
N2—C10	1.331 (4)	O5—C13	1.242 (3)
N2—C11	1.357 (3)	O2W—H2A	0.8511
N1—C1	1.324 (3)	O2W—H2B	0.8494
C4—C3	1.399 (4)	C10—H10	0.9300
C4—C5	1.442 (4)	O3W—H3B	0.8496
C11—C7	1.406 (4)	O3W—H3A	0.8513
O1—C13	1.267 (3)	O1W—H1A	0.8500
O2—C14	1.433 (3)	O1W—H1B	0.8500
O2—H21	0.8519		
			
C16—O3—Zn1^i^	120.39 (17)	C14—O2—Zn1	111.16 (15)
O6—C16—O3	124.6 (3)	C14—O2—H21	111.1
O6—C16—C15	117.8 (3)	Zn1—O2—H21	119.0
O3—C16—C15	117.3 (2)	C6—C5—C4	121.4 (2)
C15—O4—Zn1^i^	112.24 (15)	C6—C5—H5	119.3
C15—O4—H22	106.9	C4—C5—H5	119.3
Zn1^i^—O4—H22	117.1	N1—C1—C2	122.8 (2)
O4—C15—C14	113.2 (2)	N1—C1—H1	118.6
O4—C15—C16	109.1 (2)	C2—C1—H1	118.6
C14—C15—C16	113.1 (2)	C2—C3—C4	119.6 (2)
O4—C15—H15	107.0	C2—C3—H3	120.2
C14—C15—H15	107.0	C4—C3—H3	120.2
C16—C15—H15	107.0	C5—C6—C7	121.5 (2)
O3^ii^—Zn1—O1	99.98 (9)	C5—C6—H6	119.2
O3^ii^—Zn1—N1	160.80 (9)	C7—C6—H6	119.2
O1—Zn1—N1	93.98 (8)	O2—C14—C13	108.1 (2)
O3^ii^—Zn1—N2	92.56 (9)	O2—C14—C15	112.6 (2)
O1—Zn1—N2	159.34 (9)	C13—C14—C15	112.7 (2)
N1—Zn1—N2	78.22 (8)	O2—C14—H14	107.8
O3^ii^—Zn1—O4^ii^	76.09 (7)	C13—C14—H14	107.8
O1—Zn1—O4^ii^	92.31 (8)	C15—C14—H14	107.8
N1—Zn1—O4^ii^	90.32 (8)	C3—C2—C1	119.6 (2)
N2—Zn1—O4^ii^	106.70 (9)	C3—C2—H2	120.2
O3^ii^—Zn1—O2	90.46 (8)	C1—C2—H2	120.2
O1—Zn1—O2	75.15 (7)	C11—C7—C8	117.0 (3)
N1—Zn1—O2	105.92 (9)	C11—C7—C6	118.5 (3)
N2—Zn1—O2	88.49 (8)	C8—C7—C6	124.5 (3)
O4^ii^—Zn1—O2	159.91 (7)	C9—C8—C7	120.1 (3)
N1—C12—C4	122.8 (2)	C9—C8—H8	119.9
N1—C12—C11	117.4 (2)	C7—C8—H8	119.9
C4—C12—C11	119.8 (2)	H4A—O4W—H4B	105.1
H5A—O5W—H5B	139.5	C8—C9—C10	119.8 (3)
C10—N2—C11	118.5 (2)	C8—C9—H9	120.1
C10—N2—Zn1	128.0 (2)	C10—C9—H9	120.1
C11—N2—Zn1	113.43 (16)	H6B—O6W—H6A	89.5
C1—N1—C12	117.8 (2)	O5—C13—O1	124.1 (2)
C1—N1—Zn1	128.78 (17)	O5—C13—C14	117.3 (2)
C12—N1—Zn1	113.42 (15)	O1—C13—C14	118.6 (2)
C3—C4—C12	117.4 (2)	H2A—O2W—H2B	105.1
C3—C4—C5	124.0 (2)	N2—C10—C9	122.0 (3)
C12—C4—C5	118.7 (2)	N2—C10—H10	119.0
N2—C11—C7	122.6 (2)	C9—C10—H10	119.0
N2—C11—C12	117.4 (2)	H3B—O3W—H3A	107.4
C7—C11—C12	120.0 (2)	H1A—O1W—H1B	109.9
C13—O1—Zn1	118.07 (17)		

Symmetry codes: (i) *x*−1, *y*, *z*; (ii) *x*+1, *y*, *z*.

### Hydrogen-bond geometry (Å, °)

**Table:**

*D*—H···*A*	*D*—H	H···*A*	*D*···*A*	*D*—H···*A*
O1W—H1A···O5W^iii^	0.85	1.99	2.787 (3)	157
O2W—H2A···O3W	0.85	2.05	2.819 (4)	150
O2W—H2B···O6W^iv^	0.85	1.98	2.822 (4)	172
O3W—H3A···O5^v^	0.85	2.17	2.802 (3)	131
O3W—H3B···O1W^vi^	0.85	1.93	2.776 (4)	177
O4W—H4A···O5W^iii^	0.85	2.00	2.789 (4)	154
O4W—H4B···O6^vii^	0.85	1.99	2.718 (3)	143
O5W—H5A···O3^v^	0.85	2.04	2.880 (3)	168
O5W—H5B···O1	0.85	2.10	2.909 (3)	160
O6W—H6A···O1W^viii^	0.85	2.02	2.816 (4)	155
O6W—H6B···O5	0.85	2.04	2.886 (3)	174
O2—H21···O2W^ix^	0.85	1.82	2.655 (3)	164
O4—H22···O4W^x^	0.85	1.75	2.599 (3)	178

Symmetry codes: (iii) −*x*+2, *y*+1/2, −*z*+1/2;  (iv) −*x*+3/2, −*y*+1, *z*−1/2; (v) −*x*+1, *y*+1/2, −*z*+1/2; (vi) *x*−1/2, −*y*+3/2, −*z*; (vii) *x*, *y*+1, *z*; (viii) −*x*+2, *y*−1/2, −*z*+1/2; (ix) *x*, *y*−1, *z*; (x) −*x*+1, *y*−1/2, −*z*+1/2.
